# The Use of the Alcoholic Lotion in Phthisis Pulmonalis

**Published:** 1844-06-01

**Authors:** 


THE USE OF THE ALCOHOLIC LOTION IN PHTHISIS PULMONALIS.

Dr. M. Hall says that this lotion possesses a power in checking the progress of the deposition and softening of tubercle in the lungs, beyond any other which he ever tried. The number of patients who have recovered from incipient phthisis under its use, and who, after many years, are still living, and in apparent health, warrants him, he thinks, in making this assertion, and in expressing himself in strong terms in regard to its extreme value. It is to be constantly applied by means of six folds of linen over and across the upper lobes of the lungs. One part of pure alcohol is mixed with three parts of water. It is applied tepid at first, and afterwards of the temperature of the atmosphere. It is used in small quantity at a time, every five minutes, so that the application may always consist of alcohol and of water. (If applied in larger quantity, and less frequently, the alcohol would evaporate, and water alone be left, and this would be the source of a feeling of discomfort, instead of the glow which the alcohol induces.) The application is easily made; a piece of soft linen, of the size of a very large sheet of letter-paper, being folded in the usual manner, is then folded twice more, in lines parallel with the first, so that the whole consists of six folds. These are stretched, applied across the upper part of the thorax, just below the clavicles, and fastened to the shoulder-straps, or other part of the dress, which latter is to be arranged so as to be readily opened and closed. A sponge, the size of a walnut, is then filled with the lotion, and pressed upon the linen along its whole course, the dress being opened for this purpose, and immediately closed. The operation need not occupy five seconds; it should be repeated every five minutes. The application of the lotion should be incessant during the day, and all walking hours, the dress being light, or even entirely removed, so as to allow of free and rapid evaporation. It is to be suspended during the night. Dr. Hall mentions briefly several cases in which this lotion appeared to do good, and he begs to be understood as stating only the fact, that he has witnessed many, very many cases of incipient phthisis checked by the strenuous application of the alcoholic lotion, and the patients restored to apparent health; these cases having been proved to be phthisis by the presence of the physical signs, as well as the morbid symptoms of this dire disease. Neither does he recommend it as a cure for phthisis. He thinks it the most important remedy in this disease which we possess; but he would by no means neglect any of the other well-known aids in the treatment of phthisis. Of these, change of air, free exposure to the sea-breezes, a sea voyage, a mild climate, a chalky soil, a locality screened from the north-east winds, gentle exercises, especially on horse-back, a meat diet, with a little of Bass's ale, perhaps, but without other stimulus, the system of sponging with sea water, or salt and water, or vinegar and water, light clothing, with flannel next the skin, &c., the plan recommended by the late Dr. Stewart, &c., constitute the additional remedies to be adopted in the treatment of phthisis.—*London Lancet.*

